# Central venous-to-arterial carbon dioxide difference as a prognostic tool in high-risk surgical patients

**DOI:** 10.1186/s13054-015-0917-6

**Published:** 2015-05-13

**Authors:** Emmanuel Robin, Emmanuel Futier, Oscar Pires, Maher Fleyfel, Benoit Tavernier, Gilles Lebuffe, Benoit Vallet

**Affiliations:** Department of Anaesthesiology and Intensive Care Medicine, University Hospital of Lille, Lille, France; Department of Anaesthesiology and Intensive Care Medicine, Hospital Estaing, University Hospital of Clermont-Ferrand, Clermont-Ferrand, France

## Abstract

**Introduction:**

The purpose of this study was to evaluate the clinical relevance of high values of central venous-to-arterial carbon dioxide difference (PCO_2_ gap) in high-risk surgical patients admitted to a postoperative ICU. We hypothesized that PCO_2_ gap could serve as a useful tool to identify patients still requiring hemodynamic optimization at ICU admission.

**Methods:**

One hundred and fifteen patients were included in this prospective single-center observational study during a 1-year period. High-risk surgical inclusion criteria were adapted from Schoemaker and colleagues. Demographic and biological data, PCO_2_ gap, central venous oxygen saturation, lactate level and postoperative complications were recorded for all patients at ICU admission, and 6 hours and 12 hours after admission.

**Results:**

A total of 78 (68%) patients developed postoperative complications, of whom 54 (47%) developed organ failure. From admission to 12 hours after admission, there was a significant difference in mean PCO_2_ gap (8.7 ± 2.8 mmHg versus 5.1 ± 2.6 mmHg; *P* = 0.001) and median lactate values (1.54 (1.1-3.2) mmol/l versus 1.06 (0.8-1.8) mmol/l; *P* = 0.003) between patients who developed postoperative complications and those who did not. These differences were maximal at admission to the ICU. At ICU admission, the area under the receiver operating characteristic curve for occurrence of postoperative complications was 0.86 for the PCO_2_ gap compared to Sequential Organ Failure Assessment score (0.82), Simplified Acute Physiology Score II score (0.67), and lactate level (0.67). The threshold value for PCO_2_ gap was 5.8 mmHg. Multivariate analysis showed that only a high PCO_2_ gap and a high Sequential Organ Failure Assessment score were independently associated with the occurrence of postoperative complications. A high PCO_2_ gap (≥6 mmHg) was associated with more organ failure, an increase in duration of mechanical ventilation and length of hospital stay.

**Conclusion:**

A high PCO_2_ gap at admission in the postoperative ICU was significantly associated with increased postoperative complications in high-risk surgical patients. If the increase in PCO_2_ gap is secondary to tissue hypoperfusion then the PCO_2_ gap might be a useful tool complementary to central venous oxygen saturation as a therapeutic target.

## Introduction

There is increasing evidence that individually optimized hemodynamic therapy oriented on goals to maintain and improve tissue perfusion and/or oxygenation improves patient outcome [1]. The development of tissue hypoxia is a leading cause of postoperative organ failure and mortality following major surgery [2,3]. Early recognition and correction of warning signals of persistent inadequacy of tissue perfusion is therefore of particular importance, especially in patients with a reduced physiological reserve [1,4].

The inability to meet an increase in oxygen (O_2_) demand with surgical trauma either by an increase in O_2_ delivery or an increase in O_2_ extraction can lead to tissue hypoxia [5,6]. Several markers of impaired tissue oxygenation have been explored to help identify patients at increased risk of complications. Postoperative organ failure has been shown to be associated with reduced central venous O_2_ saturation (ScvO_2_), which explores the balance of O_2_ delivery and tissue O_2_ consumption [7]. However, there is evidence that O_2_-derived variables are poorly correlated with anaerobic metabolism [8-11]. Indeed, both normal and high values (that is, ≥75%) for ScvO_2_ do not preclude the presence of tissue hypoxia in case of impaired O_2_ extraction capabilities, which may therefore limit the usefulness of ScvO_2_ monitoring [12,13]. In contrast, it has also been shown that strategies aimed at reducing high serum lactate levels, as a warning signal of a persistent tissue hypoxia at ICU admission, could reduce length of stay and mortality [14,15]. However, a rise in lactate level may be delayed compared with markers of tissue oxygenation adequacy, such as oxygen extraction [16], and could be not sensitive enough to reflect tissue hypoperfusion [14].

Previous relatively small studies have proposed central venous-to-arterial carbon dioxide gradient (PCO_2_ gap), a global index of tissue perfusion, as a useful measurement to characterize the insufficient flow state in spite of apparently normal macrocirculatory parameters [17,18]. Tissue partial pressure of carbon dioxide (PCO_2_) reflects metabolic alterations due to inadequate perfusion in actively metabolized tissues [19]. The PCO_2_ gap, which has been shown to be inversely related to cardiac output (CO) [20], is considered as a marker of the ability of the venous blood flow to remove the CO_2_ excess produced in tissues [21]. Thus, an impaired tissue perfusion during a reduced blood flow is the main determinant of a rise of the PCO_2_ gap [22]. However, despite promising findings from both experimental and clinical data, the prognostic significance of the PCO_2_ gap has only been examined to a small extent in the context of major surgical trauma. The purpose of this study was to evaluate the clinical relevance of high values of the PCO_2_ gap, and their relationships to other markers of impaired tissue perfusion and oxygenation (that is, blood lactate and ScvO_2_). We hypothesized that the PCO_2_ gap could serve as a useful tool to help identify patients at high risk of postoperative complications at ICU admission following major surgery.

## Methods

### Patients

This was a prospective single-center observational study of patients scheduled for major abdominal and vascular surgery and admitted to the ICU of a University Hospital over a 1-year period. The study was approved by local Research Ethics Committee of the University Hospital of Lille, France, which permitted anonymous data analysis. The requirement for written inform consent was waived due to the strict observational design of this study.

Inclusion criteria adapted from Schoemaker and colleagues [23] are summarized in Table 1 and are divided into demographic, surgical and intensive care criteria. All patients undergoing abdominal or vascular surgery were included if they had one of the following criteria: 1) one demographic criterion and one surgical criterion; 2) three or more demographic criteria; 3) three or more surgical criteria; 4) one intensive care criterion.

**Table 1:** **Demographic, surgical and intensive care inclusion criteria**

**High-risk criteria**	**Values**
**Demographic criteria**	
Age ≥70 years	42 (37)
ASA class ≥3	70 (61)
Severe nutritional problems	11 (10)
Previous severe respiratory illness	24 (11)
Chronic renal failure	6 (5)
Chronic liver failure	7 (6)
Ischemic heart disease (infarction or angina)	51 (44)
Malignant neoplasia	67 (58)
**Surgical criteria**	
Major abdominal surgery	82 (71)
Prolonged surgery ≥8 hours	9 (8)
Urgent surgery	22 (19)
Septic surgery	24 (21)
Vascular clamping ≥1 hour	4 (4)
Surgical procedures	
Esophagectomy	22 (19)
Gastrectomy	9 (8)
Small bowel resection	17 (15)
Large bowel resection	20 (17)
Hepatectomy	21 (18)
Pancreatectomy	16 (14)
Intra-abdominal vascular surgery	24 (21)
Other	2 (2)
**Intensive care criteria**	
Shock	38 (33)
Acute respiratory failure	32 (28)
Hemorrhage (hemoglobin <7 g/dl)	10 (9)
Acute coronary syndrome	7 (6)

Data are presented as absolute value (%). Severe nutritional problems: body mass index ≤17 kg/m^2^ or weight loss >10% in 6 months. Chronic renal failure: creatinine clearance <60 ml/min per 1.73 m^2^ or creatinine >176 μmol/l. Chronic liver failure: bilirubin >78 μmol/l or prothrombin time <55% or well-documented cirrhosis.

ASA, American Society of Anesthesiology physical status.

### Study protocol

As part of our routine hemodynamic monitoring during major surgery, all patients were monitored with central venous (standard two-lumen catheter, Arrow, Wayne, Pennsylvania, USA; or PreSep catheter with oximetry, Edwards Lifesciences, Irvine, California, USA) and arterial (Seldicath, Plastimed, Le Plessis Bouchard, France) catheters placed before the beginning of surgery. The central venous line was positioned with the tip within the superior vena cava, and correct positioning was verified by chest radiograph. Until admission to the ICU, anesthesia and surgical procedures were performed according to the local standards. No specific hemodynamic protocol was used during surgery. All patients were admitted to the ICU immediately after surgery and were all managed according to our local standards of care.

### Data collection and outcome measures

Standard postoperative monitoring included: electrocardiograph (heart rate), invasive mean arterial pressure, pulse oxymetry and urine output. In all patients, the PCO_2_ gap, calculated as the difference between central venous partial pressure of carbon dioxide and arterial partial pressure of carbon dioxide, ScvO_2_, serum lactate level, blood gas analysis, troponin I and routine laboratory tests were obtained by intermittent blood sampling immediately after admission (H0) and repeated 6 (H6) and 12 hours (H12) later. At ICU admission, data on demographics (age, sex, weight), type of surgical procedure, American Society of Anesthesiology Physical Status score, Simplified Acute Physiology Score (SAPS) II [24], presence of catecholamine and the need for mechanical ventilation were recorded in all patients. Postoperative organ failure was assessed using the Sequential Organ Failure Assessment (SOFA) score recorded daily until ICU discharge.

Briefly, the organ failure criteria are:

- Circulatory failure: use of catecholamine to maintain a mean arterial pressure ≥65 mmHg after a suitable fluid loading.
- Acute respiratory failure: need for mechanical ventilation or noninvasive ventilation.
- Acute kidney injury: 1.5-times increase in creatinine serum level or increased creatinine >0.3 mg/dl or urine output <0.5 ml/kg per hour for 6 hours.
- Neurological impairment: stroke with focal deficit or coma (Glasgow score ≤8) or delirium.

Postoperative complications were assessed in accordance with previously defined criteria [25,26] until hospital discharge or death as follows: postoperative sepsis (pneumonia, intraperitoneal abscess, wound infection, peritonitis and urinary tract infection), acute respiratory failure, acute renal and cardiac failures, postoperative hemorrhage, ischemic events, and postoperative mortality. Patients were followed-up until hospital discharge or death.

### Statistical analysis

The study population was divided into two groups according to the occurrence of postoperative complications. Normal distribution of all variables was accessed by graphical methods and the Kolmogorov-Smirnov test. All data are presented as absolute value (%), as mean ± standard deviation or as median (interquartile range) when necessary. Differences between the two groups at baseline were analyzed using the Student’s *t* test or Mann-Whitney *U* test for continuous variables, and chi-square test or Fisher’s exact test for categorical variables. A repeated-measure analysis of variance was used to compare variables over time. When the sphericity assumption has been violated as assessed by Mauchly’s test, the degrees of freedom were corrected using Greenhouse-Geisser estimates of sphericity. A Bonferroni correction was used for *post hoc* tests. Univariate analysis was performed to test associations with postoperative complications. A logistic regression was performed for multivariate analysis for all univariate relevant covariates that discriminate between the two groups (*P* value <0.05 was set as the limit for inclusion). A hierarchical entry method in two blocks was used. In the first block, variables usually known to influence prognosis were entered. In the second block, all other variables were entered. Receiver operating characteristic (ROC) curves were generated to identify optimal cut-off values for outcome associations, and the area under the ROC curve, sensitivity and specificity were calculated. The optimal threshold value from the ROC curves was assessed to obtain the highest Youden index and positive likelihood ratio. A *P* value less than 0.05 was considered statistically significant. Statistical analysis was performed using the SPSS 17.0 software (Chicago, IL, USA).

## Results

Between May 2008 and May 2009, 115 patients who fulfilled the entry criteria were included in the study. Baseline characteristics of the study population are given in Table 1. The median American Society of Anesthesiology Physical Status score was 3.0 (2.0-3.0), mean age was 65 ± 12 years, and 75% were male. At the time of inclusion (T0), the median SAPS II score was 19.5 (15.0-28.7) and the mean ScvO_2_ and PCO_2_ gap were 77.3 ± 6.3% and 7.2 ± 3.3 mmHg, respectively. A total of 43% of patients were mechanically ventilated, and 36% received catecholamine infusion. The median duration of mechanical ventilation was 0.0 (0.0-3.0) days. The SOFA scores were 4.0 (1.0-10.0), 4.0 (1.0-8.0), and 4.0 (1.0-8.0) at postoperative days 1, 2 and 3, respectively. The median ICU and hospital length of stays were 6.0 (4.0-8.0) days and 21.0 (15.0-29.0) days, respectively. The 28-day mortality rate was 8% (septic shock, n = 4; acute mesenteric ischemia, n = 2; myocardial infarction, n = 2; massive acute blood loss, n = 1).

### Association with outcome

**Figure 1:**
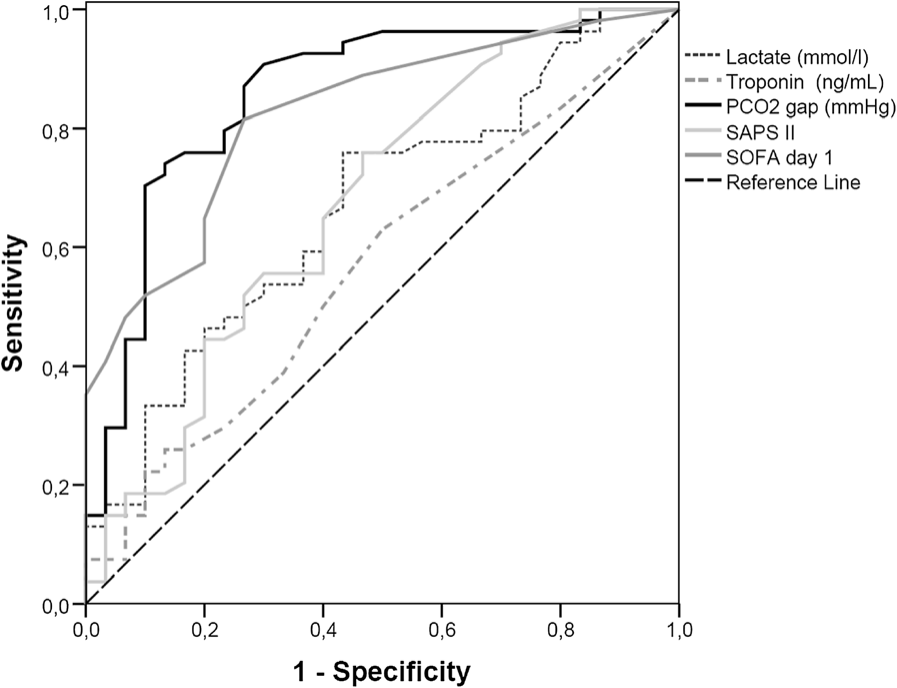
Discriminant factors of postoperative complications. Receiver operating characteristic curve comparing the ability of central venous-to-arterial difference in carbon dioxide (PCO_2_ gap), Sequential Organ Failure Assessment (SOFA) score, Simplified Acute Physiology Score (SAPS) II score, lactate level and troponin level at baseline to discriminate between patients who did (n = 78) and did not (n = 37) develop postoperative complications. Areas under the curve are 0.86; 0.82; 0.67; 0.67 and 0.57, respectively.

A total of 78 (68%) patients developed postoperative complications during their ICU stay, of whom 57 (50%) patients developed postoperative sepsis and 54 (47%) patients developed organ failure (Table 2). At the time of surgery, patients with postoperative complications were more likely to undergo urgent surgery (Table 3). There were no other statistically significant differences in baseline high-risk criteria between the two patient groups. Patients with postoperative complications were more severely ill on ICU admission (SOFA score: 7.0 (3.0-12.0) versus 1.0 (1.0-2.5), *P* < 0.001; SAPS II score: 23.0 (16.5-31.2) versus 15.5 (12.0-24.2), *P* = 0.008), had longer durations of mechanical ventilation (2.0 (0.0-3.0) days versus 0.0 (0.0-0.0) days, *P* < 0.001) and longer durations of hospital stay (25.0 (20.0-34.0) days versus 14.0 (12.0-160), *P* < 0.001). On the day of ICU admission, there were statistically significant differences in lactate level (*P* = 0.006), but not in ScvO_2_ values (*P* = 0.17) between patients who did and did not develop postoperative complications. Univariate analysis identified nine variables on ICU admission associated with the occurrence of postoperative complications on ICU admission: lactate level (*P* = 0.006), troponin level (*P* = 0.025), bicarbonate level (*P* = 0.008), arterial O_2_ saturation (*P* = 0.026), urine output (*P* = 0.023), PCO_2_ gap value (*P* < 0.001), SAPS II (*P* = 0.008), SOFA score (*P* < 0.001) and emergency surgery (*P* = 0.04). Multivariate analysis showed that a high PCO_2_ gap (odds ratio = 1.93, 95% confidence interval (CI) 1.36 to 2.75, *P* < 0.001) and a high SOFA score (odds ratio = 1.52 95% CI 1.14 to 2.02, *P* = 0.004) at H0 were independently associated with the occurrence of postoperative complications (Table 4). The same results were observed at H6 (data not shown). The area under the ROC curve for the occurrence of postoperative complications was 0.86 (95% CI 0.77 to 0.95) for the PCO_2_ gap. The area under the ROC curve for organ failure for SOFA score, SAPS II score, lactate level and troponin value were 0.82, 0.67, 0.67 and 0.57, respectively (Figure 1). The optimal PCO_2_ gap value on ICU admission was 5.8 mmHg (sensitivity 90.7%, specificity 70.0%, positive predictive value 86.6%, and negative predictive value 78.8%) for discriminating between patients who did and patients who did not develop postoperative complications. Of the 54 patients who developed organ failure, 46 had a PCO_2_ gap ≥6 mmHg. A high PCO_2_ gap (>6 mmHg) was observed in 68% of the patients upon admission to the ICU after surgery. Compared with patients with a low PCO_2_ gap on ICU admission, a high PCO_2_ gap was associated with more organ failure (*P* < 0.001), and an increase in duration of mechanical ventilation (*P* = 0.002) and length of hospital stay (*P* < 0.001) (Table 5). In addition, a high PCO_2_ gap was associated with a higher 28-day mortality rate (11.5% versus 0%, *P* = 0.056).

**Table 2:** **Postoperative complications**

**Variables**	
Sepsis	57 (49.6)
Pneumonia	37 (32.2)
Peritonitis	17 (14.8)
Wound infection	2 (1.7)
Urinary tract infection	1 (0.8)
Acute renal failure	18 (15.7)
Acute cardiac failure	10 (8.7)
Acute myocardial infarction	5 (6.1)
Pulmonary embolism	3 (2.6)
Hemorrhage	14 (12.2)
Lower limb ischemia	11 (9.6)

Data are presented as absolute values (%).

**Table 3:** **Baseline characteristics of patients who did and did not develop postoperative complications**

**Variables**	**Patients with postoperative complications**	**Patients without postoperative complications**	***P*** **value**
	**(n = 78)**	**(n = 37)**	
**Severity scores**			
ASA class	3.0 (2.0-3.0)	3.0 (2.0-3.0)	0.16
ASA class ≥3 (%)	51 (65)	19 (51)	0.150
SOFA	7.0 (3.0-12.0)	1.0 (1.0-2.5)	**<0.001**
SAPS II	23.0 (16.5-31.2)	15.5 (12.0-24.2)	**0.008**
**High-risk criteria** (%)			
Age, years	64 ± 13	65 ± 10	0.84
Age ≥70 years	30 (38)	12 (32)	0.53
Severe nutritional problems	8 (10)	3 (8)	1.00
Previous respiratory illness	20 (26)	4 (11)	0.07
Chronic renal failure	5 (6)	1 (3)	0.66
Chronic liver failure	6 (8)	1 (3)	0.43
Ischemic heart disease	32 (41)	19 (51)	0.30
Malignant neoplasia	46 (59)	21 (57)	0.82
Major abdominal surgery	52 (67)	30 (81)	0.11
Prolonged surgery ≥8 hours	6 (8)	3 (8)	1.00
Urgent surgery	19 (24)	3 (8)	**0.04**
Septic surgery	19 (24)	5 (13)	0.18
Vascular clamping ≥1 hour	4 (5)	0 (0)	0.30
**Physiological parameters**			
Mean arterial pressure, mmHg	82 ± 16	85 ± 14	0.30
Urine output, ml/3 hours	266 ± 228	345 ± 258	**0.023**
Biologic parameters			
Serum lactate, mmol/l	1.54(1.1-3.2)	1.06 ± (0.8-1.8)	**0.006**
Serum bicarbonate, mmol/l	19.6 ± 4.2	21.4 ± 2.7	**0.008**
Hemoglobin, g/dl	10.4 ± 1.8	10.8 ± 1.7	0.39
Troponin I, ng/ml	0.03 (0.01-0.11)	0.01 (0.00-0.04)	**0.025**
Arterial pH	7.33 ± 0.08	7.35 ± 0.07	0.47
Venous pH	7.28 ± 0.09	7.30 ± 0.06	0.06
PCO_2_ gap, mmHg	8.7 ± 2.8	5.1 ± 2.6	**<0.001**
PcvCO_2_, mmHg	46.1 ± 6.7	45.9 ± 6.0	0.92
PaCO_2_, mmHg	37.4 ± 6.5	40.7 ± 6.2	0.09
ScvO_2_, %	76.3 ± 6.3	78.0 ± 5.2	0.17
SaO_2_, %	99.2 (98.4-99.5)	98.8 (98.1-99.2)	**0.026**
PaO_2_, mmHg	145 (116–175)	135 (110–156)	0.05
PcvO_2_, mmHg	46.0 (41.7-54.0)	48,0 (40.5-52.0)	0.77

Data are presented as absolute values (%), mean ± standard deviation, or median (interquartile range). Comparison between groups were assessed by Student’s *t* test or Mann-Whitney *U* test when necessary. Significant *P* values are indicated in bold text. ASA, American Society of Anesthesiology physiological status; PaCO_2_, arterial partial pressure of carbon dioxide; PaO_2_, arterial partial pressure of oxygen; PCO_2_ gap, central venous-to-arterial carbon dioxide gradient; PcvCO_2_, central venous partial pressure of carbon dioxide; PcvO_2_, central venous partial pressure of oxygen; SaO_2_, arterial oxygen saturation; SAPS, Simplified Acute Physiology Score; SOFA, Sequential Organ Failure Assessment; ScvO_2_, central venous oxygen saturation.

**Table 4:** **Logistic regression results: variables associated with the occurrence of postoperative complications**

	**B (SE)**	**Odds ratio**	**95% confidence interval**	***P***
Constant	−5.61 (3.50)			
PCO_2_ gap	0.66 (0.18)	1.93	1.36-2.75	**<0.001**
SOFA score	0.42 (0.15)	1.52	1.14-2.02	**0.004**
Lactate	0.38 (0.37)	1.47	0.71-3.02	0.300
SAPS II score	0.04 (0.04)	1.04	0.96-1.13	0.347
Emergency surgery	0.09 (1.17)	1.10	0.11-10.80	0.937
Bicarbonate	−0.07 (0.11)	0.931	0.75-1.16	0.931
Troponin	1.87 (1.47)	6.50	0.36-117.06	0.204
diuresis	0.0003 (0.002)	1.00	0.997-1.003	0.825

Model χ^2^ = 54.96, *P* < 0.001, R^2^ = 0.50 (Hosmer and Lemeshow), R^2^ = 0.48 (Cox and Snell), R^2^ = 0.66 (Nagelkerke). Significant *P* values are indicated in bold text. PCO_2_ gap, central venous-to-arterial difference in carbon dioxide; SAPS II, Simplified Acute Physiology Score II; SE, standard error; SOFA, Sequential Organ Failure Assessment.

**Table 5:** **Outcome of patients with high and low values of PCO**_**2**_ **gap on ICU admission**

**Variables**	**PCO2 gap ≥6 mmHg**	**PCO2 gap <6 mmHg**	***P*** **value**
	**(n = 78)**	**(n = 37)**	
Total duration of MV, days	2.0 (0.0-3.2)	0.0 (0.0-0.0)	**<0.001**
Length of ICU stay, days	6.0 (4.0-8.2)	5.0 (4.0-7.5)	0.287
Length of hospital stay, days	22.5 (17.0-32.2)	16.0 (13.0-23.5)	**0.002**
Organ failure	46 (59.0%)	8 (21.6%)	**< 0.001**
28-day mortality	9 (11.5%)	0	0.056

Areas under the curve are 0.86; 0.82; 0.67; 0.67 and 0.57, respectively. Data are presented as medians (interquartile range) or absolute value (%). Significant *P* values are indicated in bold text. MV, mechanical ventilation; PCO_2_ gap, central venous-to-arterial difference in carbon dioxide.

### Trends in PCO_2_ gap

**Figure 2:**
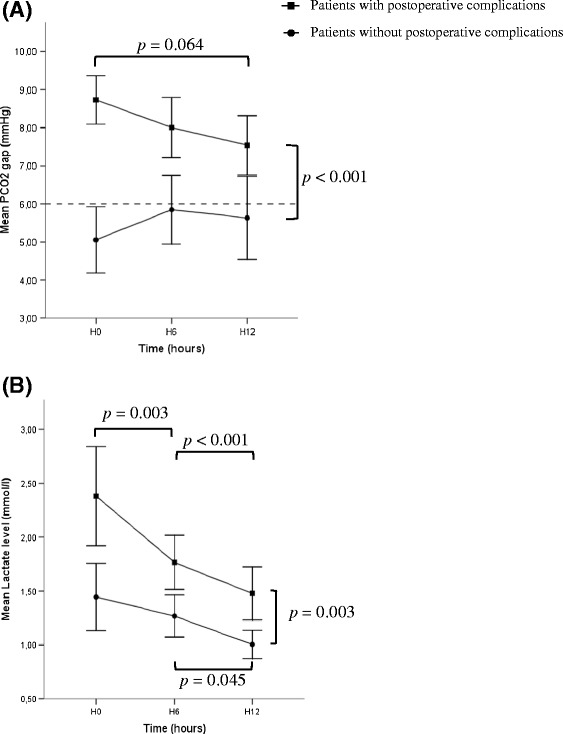
Trends in PCO_2_ gap and lactate level. **(A)** Trends in PCO2 gap (mmHg) and **(B)** trends in lactate level (mmol/l) in patients who did (n = 78; square markers) and did not (n = 37; circle markers) develop postoperative complications. PCO_2_ gap, central venous-to-arterial difference in carbon dioxide.

**Figure 3:**
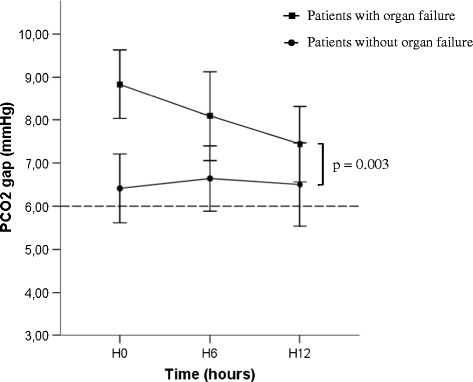
Trends in PCO_2_ gap and organ failure. Trends in PCO_2_ gap (mmHg) in patients who developed organ failure (n = 54; square markers) and those who did not (n = 61; circle markers). Results are expressed as means ± 95% confidence interval. PCO_2_ gap, central venous-to-arterial difference in carbon dioxide.

Changes in the PCO_2_ gap and lactate values during the first 12 hours are shown in Figure 2. From H0 to H12, there was a significant difference for mean PCO_2_ gap (*P* = 0.001) and mean lactate values (*P* = 0.003) between patients who did or did not develop postoperative complications. Maximal difference was present immediately after inclusion just after surgery (8.7 ± 2.8 mmHg versus 5.1 ± 2.6 mmHg, *P* < 0.001). There was a trend towards a decreased PCO_2_ gap all along the first 12 hours of medical support in the ICU for patients with postoperative complications (*P* = 0.064). Similar trends were present for the lactate level. There was also a significant difference for mean PCO_2_ gap (*P* = 0.003) between patients who developed organ failure and those who did not (Figure 3).

## Discussion

The main finding of our study is that a PCO_2_ gap >6 mmHg at ICU admission following major surgery is predictive of postoperative complications in high-risk surgical patients. Patients with an enlarged PCO_2_ gap had more organ failure, increased durations of mechanical ventilation as well as length of hospital stay, and a trend towards higher mortality rates, although the latter did not reach statistical significance.

To the best our knowledge, this study is the first to evidence the prognostic significance of an enlarged PCO_2_ gap at ICU admission in high-risk surgical patients. In patients who developed postoperative complications, the increase in PCO_2_ gap was maximal immediately after ICU admission and gradually decreased thereafter as a result of medical support. The diagnostic performance of the PCO_2_ gap is quite similar to the SOFA score with the huge advantage of being measurable at patient admission. In addition, the measurement of the PCO_2_ gap is much more responsive than the SOFA score and easy to implement at the bedside. These results are supported by the results of a previous study by our group in which an enlarged PCO_2_ gap was associated with an increased rate of postoperative complications in patients who remained inadequately managed by volume loading during an individualized goal-directed therapy [17]. These results also echo those of previous studies in patients with severe sepsis or septic shock in which a large PCO_2_ gap was associated with higher rates of organ failure and greater mortality [18,21,27]. In all these studies, the thresholds for PCO_2_ gap values were around 5 to 6 mmHg, as in our study.

The increase in venous PCO_2_ would reflect a state of insufficient flow relative to CO_2_ production [28,29]. Indeed, in an *in situ*, vascularly isolated, innervated dog hindlimb model, Vallet and colleagues evidenced that the PCO_2_ gap increased during low blood flow-induced tissue hypoxia (ischemic hypoxia) while it remained unchanged during hypoxemia-induced hypoxia (hypoxic hypoxia) [22]. These results were confirmed in a mathematical analysis model [30] and in *in vivo* conditions in pig [31] and in sheep [9]. These results are also in agreement with those of Bakker and colleagues [21] who showed that, in patients with septic shock, the PCO_2_ gap was smaller in survivors than in non-survivors, despite quite similar CO, O_2_ delivery (DO_2_) and O_2_ consumption (VO_2_) values. In septic shock patients, characterized by an increased PCO_2_ gap and a low flow state, fluid challenge was found to lower the PCO_2_ gap while increasing CO [32]. In contrast, no significant changes in CO and PCO_2_ gap were found in patients with normal PCO_2_, thus confirming the relationship between an increased PCO_2_ gap and insufficient flow [32].

In our study, ScvO_2_ did not allow us to discriminate between patients with and without postoperative complications. These results seem to contradict previous studies. Indeed, recently published data clearly demonstrate that low ScvO_2_ during and after major abdominal surgery is associated with an increased risk of postoperative complications [7,16,33]. In addition, ScvO_2_ was part of early goal-directed therapy protocol algorithms that have proven their effectiveness in improving the prognosis of patients [16,34]. As the use of ScvO_2_ has become increasingly popular in the management of high-risk surgical patients, one part of our patients (at the convenience of the anesthetist in charge of the patient) had already been treated using ScvO_2_ during surgery before inclusion in the study. The hemodynamics of our patients were in part optimized, as evidenced by ScvO_2_ values above 70% in both groups. Another point to consider is that sepsis was the main cause of postoperative complications in our study (47%). In this situation where microcirculation failure is frequent, a normal or high ScvO_2_ value does not preclude tissue hypoperfusion [12,13,35]. According to the modified Fick equation applied to CO_2_, the PCO_2_ gap is linearly related to CO_2_ production (VCO_2_) and inversely related to CO [29]. In situations where the VO_2_/DO_2_ relationship is satisfied, the flow is sufficient to wash out the CO_2_ produced by the tissue even if there is an additional anaerobic VCO_2_ [22]. Conversely, when blood flow is low, the PCO_2_ gap may increase even if there is no increase in VCO_2_ [31]. Taken together, these factors may explain why, in some of our patients, the PCO_2_ gap was increased while ScvO_2_ was normal and ScvO_2_ failed to predict postoperative complications [36].

Similarly, lactate levels were not an independent factor associated with postoperative complications, unlike the PCO_2_ gap. This difference is not entirely a surprise since our surgical patients benefited from immediate hemodynamic support in the operating room and intensive care. Therefore, these patients were not necessarily in a decompensated state as evidenced by the small increase in lactate levels (<2.5 mmol/l on average) and ScvO_2_ > 70% including patients who present with postoperative complications. The increase in PCO_2_ gap seems only to suggest that there is a hemodynamic optimization margin for these patients. Moreover, the PCO_2_ gap and lactate levels may reflect different events since lactate clearance is slower than the dynamic and rapid change in PCO_2_ gap; the lactate level could reflect the hemodynamic state in the last hours of surgery. If there was a significant relationship between the rate of lactate at H0 and intraoperative variables, such as intravenous fluids, blood loss, episodes of low mean blood pressure ≤60 mmHg for more than 10 minutes, and duration of surgery (data not shown), the strength of this association is quite relative, since the correlation coefficients ranged from 0.273 to 0.359. If intraoperative events influenced the lactate levels at postoperative ICU admission, they were not the only explanation.

In this context, when early goal-directed therapy has reached its objectives including ScvO_2_ > 70%, the PCO_2_ gap could be a useful additional tool to continue processing hemodynamic optimization. In several studies using a goal-directed therapy in sepsis, it was demonstrated that either lactate clearance or PCO_2_ gap could be useful for identifying a persistent tissue hypoperfusion even when ScvO_2_ goals had been achieved [15,18]. In surgical patients, it has been shown that an individualized preload-targeted fluid loading to maintain tissue perfusion was not sufficient to prevent significant differences in outcome [37]. Interestingly, the mean PCO_2_ gap was larger in patients with complications with a “normalized” DO_2_/VO_2_ ratio (ScvO_2_ ≥ 71%) than in patients without complications, with 5 mmHg as the best threshold value. Associated with these previous studies, our results confirm that the PCO_2_ gap is a useful and additional tool to detect persistent tissue hypoperfusion. Moreover, the increase in lactate level, another marker of inadequate VO_2_/DO_2_ relationship, is often delayed compared to other markers such as ScvO_2_ [16]. In our study the elevation of the PCO_2_ gap was very early, starting at patient inclusion. Part of this increase was probably secondary to the intraoperative hemodynamic situation. The PCO_2_ gap at H0 was significantly higher in patients undergoing intraoperative catecholamine (6.88 ± 3.16 versus 3.02 ± 8.7, *P* = 0.006), but this effect appears to be limited to the most seriously ill patients (those receiving catecholamines) since there was no correlation between PCO_2_ gap at H0 and other intraoperative macrocirculatory variables (mean arterial pressure, heart rate, blood loss, fluid loading, blood transfusions, dieresis; data not shown).

Our study has several limitations. First, this was a single-center study involving patients undergoing major abdominal surgery. It is therefore uncertain whether our findings can be extrapolated to other non-abdominal surgery. Second, we are aware that the number of patients was relatively small which could limit the external validity of the study, and that complementary data are needed to confirm the result. Nevertheless, when we considered that one measurement of PCO_2_ ≥ 6 mmHg at inclusion was associated with the occurrence of postoperative complications, we found a *post-hoc* power >90%. Third, the use of central venous-to-arterial PCO_2_ difference as a surrogate for mixed venous PCO_2_ gap might be a further limitation. Nevertheless, it has been found that central venous PCO_2_, obtained from a simple central blood sample instead of a pulmonary arterial blood sample, is a valuable alternative to mixed PCO_2_ and that correlation with CO still exists in this context [38].

## Conclusion

This is the first study concerning the usefulness of PCO_2_ gap in high-risk surgical patients at admission in postoperative ICU confirming previous results established during a surgical period or in septic patients. There is strong support for the use of goal-directed therapy, particularly for fluid resuscitation, with ScvO_2_ as the cornerstone of these algorithms. However, once these objectives are achieved, the PCO_2_ gap might be a useful and complementary tool to detect persistent tissue hypoperfusion that could be included as an additional step in the algorithms of early goal-directed therapy protocols. As the design of our study did not formally link the changes in PCO2 gap with tissue hypoperfusion or therapeutic change, further studies are needed to confirm these findings and be extended to other forms of surgery.

## Key messages

- High PCO_2_ gap values were associated with a higher rate of postoperative complications in high-risk surgical patients.
- Threshold value is 6 mmHg.
- In further studies, PCO_2_ gap could be integrated as an additional step in the algorithms of goal-directed therapy.

## Abbreviations

CI: confidence interval
CO: cardiac output
CO_2_: carbon dioxide
DO_2_: oxygen delivery
H: hours
O_2_: oxygen
PCO_2_: partial pressure of carbon dioxide
PCO_2_ gap: central venous-to-arterial difference in carbon dioxide
ROC: receiver operating characteristic
SAPS: Simplified Acute Physiology Score
ScvO_2_: central venous oxygen saturation
SOFA: Sequential Organ Failure Assessment
VCO_2_: carbon dioxide production
VO_2_: oxygen consumption
